# Sloughing of the Internal Carotid Artery Caused by a Red-hot Poker

**Published:** 1848-08

**Authors:** 


Mr. Pollock exhibited a specimen of *Sloughing of the internal carotid artery caused by a red-hot poker.*—A man, ætat. 24, admitted into St. George's Hospital, under Mr. Cutler, on the 22nd of February, 1848.

On the 20th, while intoxicated, he placed the red-hot end of a poker between his teeth for some wager; whilst holding it he over-balanced himself, and falling forwards, the end of the poker was forced back into his mouth.

On his admission at half-past six p. m., the right tonsil appeared very sloughy, and externally there was much swelling on the right side below the jaw, and down to the neck; great difficulty in swallowing, and some dyspnœa. About ten o'clock the same evening, profuse hæmorrhage from the mouth, so that he was nearly suffocated; there was some oozing during the night. After the hæmorrhage, the pulse was hardly perceptible.

23rd. Coughed a good deal at intervals, and expectorated several clots of blood. About twelve o'clock in the day, there was another alarming gush of blood, which produced a cadaveric paleness of the face. At half past one p. m., a ligature was placed round the right carotid artery; there was hardly any blood lost on the occasion, but the pulse continued rapid, and he died at haf-past five p. m.

On examination of the body, it was found that the poker had passed through the right tonsil towards its upper part, slightly implicating the uvula on the right side; that it had produced a considerable sloughing cavity, which extended slightly outwards and backwards, and on passing the finger into this wound, the extremity of the transverse process of the second cervical vertebra was felt exposed. Tracing the branches of the right carotid upwards, it was found that the internal carotid ran along the wall of the cavity that had been occasioned by the red-hot poker, the portion of the artery next to the cavity being covered wtth sloughy tissues; but towards its upper part, and just previous to its entering the carotid canal, the inner surface of the artery was more exposed, and two small openings existed on this exposed part, one being above the other, but both close together, the coats of the artery at this point being of a sloughy appearance. The vessel was empty; the jugular vein was collapsed and empty; the ligature had been applied effectually on the common carotid, just below the point where it is crossed by the omo-hyoideus muscle; above and below the ligature there was a small coagulum. There was much œdema and effused blood in the upper part and right side of the neck, and behind the jaw; together with much œdema of the fauces and upper part of the throat.

